# A moldable sustained release bupivacaine formulation for tailored treatment of postoperative dental pain

**DOI:** 10.1038/s41598-018-29696-w

**Published:** 2018-08-15

**Authors:** Sarah D. Shepherd, Sandra C. O’Buckley, James M. Harrington, Laura G. Haines, Ginger D. Rothrock, Leah M. Johnson, Andrea G. Nackley

**Affiliations:** 1 RTI International, 3040 Cornwallis Rd, Research Triangle Park, NC 27709 USA; 2 Department of Anesthesiology, Duke University School of Medicine, 905 South LaSalle, Street #1010, Durham, NC 27710 USA; 3 Department of Endodontics, University of North Carolina, 385 South Columbia Street, St, Chapel Hill, NC 27599 USA

## Abstract

A moldable and biodegradable dental material was designed for customized placement and sustained delivery of bupivacaine (BP) within an extraction cavity. Microparticles comprising poly(lactic-co-glycolic acid) (PLGA) containing BP were generated via solvent-evaporation and combined with absorbable hemostat Gelfoam®. Kinetics of drug release were evaluated by *in vitro* dialysis assays, showing higher release within the first 24 hours, with subsequent tapering of release kinetics. Formulations of Gelfoam® and BP-PLGA microparticles (GelBP), with three targeted dosing profiles (0.25, 0.5, and 1 mg/kg/day), were evaluated alongside acute subcutaneous BP injections (2 mg/kg) to determine analgesic efficacy in a rat model of tooth extraction pain. Molar extraction resulted in mechanical and thermal cold hyperalgesia in male and female rats. GelBP outperformed acute BP in blocking post-surgical dental pain, with the 0.25 mg/kg GelBP dose preventing hypersensitivity to mechanical (p < 0.01) and thermal cold stimuli (p = 0.05). Molar extraction also resulted in decreased food consumption and weight. Males receiving acute BP and 0.25 mg/kg GelBP maintained normal food consumption (p < 0.002) and weight (p < 0.0001) throughout 7 days. Females, receiving 0.25 mg/kg GelBP maintained weight on days 5–7 (p < 0.04). Customized, sustained release formulation of anesthetic within a tooth extraction cavity holds potential to eliminate post-operative dental pain over several days.

## Introduction

The management of post-operative dental pain is imperative to eliminate needless patient suffering, improve quality of life, and reduce healthcare costs resulting from additional clinical visits^1^. Post-surgical dental pain is typically treated with orally-administered analgesics that block inflammatory mediators (e.g., non-steroidal anti-inflammatory drugs, corticosteroids) or block central mechanisms of pain perception (e.g., opioids)^2^. Although extremely beneficial for managing pain^3^, these orally delivered drugs require continuous self-medication, and hold potential for adverse systemic effects, including sleep disruption, nausea, neurological dysfunction, circulatory/respiratory depression, and addiction^2^. Alternatively, anesthetic drugs that block peripheral nerve impulses, such as bupivacaine (BP), are delivered locally to the surgical site, thereby lessening the quantity of necessary postoperative analgesics and mitigating the deleterious effects of oral medications. However, local anesthetics have a short duration, with most drugs lasting less than eight hours^4^. For instance, a single injection of BP results in a maximum plasma drug concentration within 30–45 minutes, with complete elimination after 6 hours^4^. Sustained delivery of local anesthetics may improve dental pain management by prolonging drug effectiveness, and reducing toxicity by slowing drug uptake into systemic circulation^5,6^. Current approaches to achieve sustained duration of anesthetic involve co-formulation of drug with a biocompatible polymeric carrier, which controls drug release kinetics^7^. Several extended release formulations have received FDA-approval, including the BP-liposomal injection EXPAREL®^6,8^, and the dermal anesthetic topical creams Emla® (lidocaine/prilocaine) and SonoPrep® (lidocaine)^9^.

**Figure 1:**
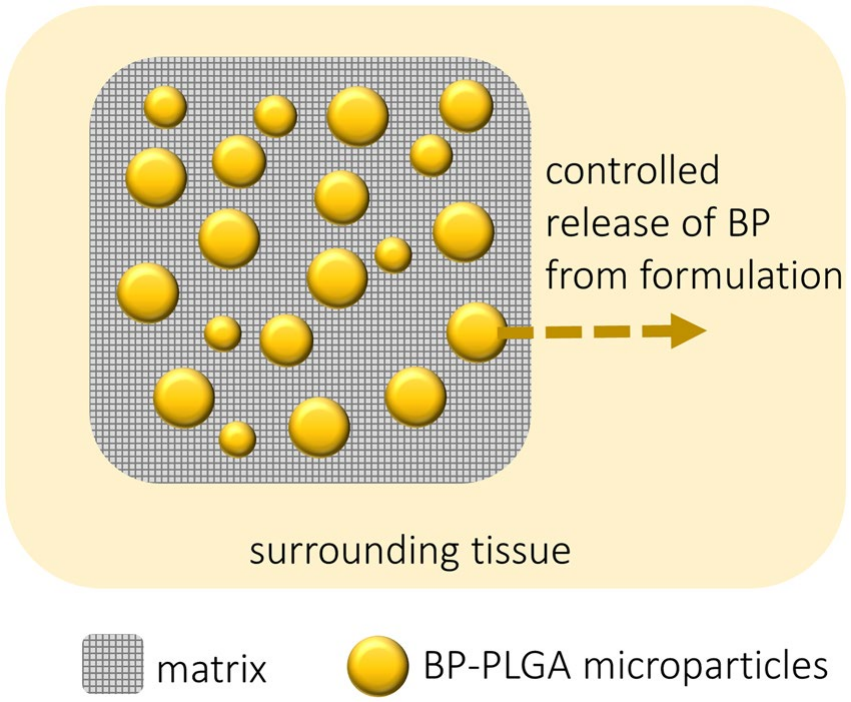
Schematic showing sustained release formulations for management of dental pain. The formulation, comprising BP-PLGA microparticles embedded in a hemostatic matrix, is placed within a dental cavity. BP is controllably released from the PLGA microparticles, diffusing through the hemostat matrix, and entering the surrounding tissue.

Treatment of post-operative dental pain following tooth extraction requires a formulation that simultaneously serves as a hemostatic agent as well as a vehicle for controlled anesthetic delivery. Current pill or injectable/implantable anesthetic delivery systems are incapable of serving as a hemostat for packing a dental cavity. Similarly, hemostatic agents currently in the clinic do not offer the capability to deliver pain medications^10^. Here, we sought to design a pain management system that combines a moldable hemostatic agent with anesthetic drug to control dental pain and support healing after a tooth extraction^11^. In particular, this controlled release formulation comprises BP- poly(lactic-co-glycolic acid) (PLGA) microparticles, embedded within a polymeric hemostatic matrix, in the form of a moldable putty. Formulations that exhibited sustainable BP release over three days *in vitro* were selected for subsequent pre-clinical assessment in a rodent model of tooth extraction. As the cavity heals, the polymer and matrix dissolve, releasing the BP continuously. By coordinating the BP release kinetics with post-operative healing, pain can be controlled for days following surgery (Fig. 1).

## Methods

### Preparation and characterization of microparticles

To enable methylene chloride dissolution of bupivacaine hydrochloride monohydrate (BP-HCl) for the solvent-evaporation method, it was converted to the soluble base form using an alkaline precipitation and filtration procedure^12^ (Supplemental Method 1). Conversion results were evaluated via X-Ray powder diffraction (XRD) (Supplemental Method 2 and Supplemental Fig. 1). The PLGA microparticles were prepared using an emulsion-solvent evaporation method^13^ (Supplemental Method 3), with the resultant emulsion added to 200 mL of 100 mM Trizma-buffered diH_2_O, and swirled to combine. The microparticles were introduced into a Buchi rotary evaporator at 45 °C for approximately 20 minutes, ramping from 1000 mbar to 215 mbar, extracting the methylene chloride. The microparticles were centrifuged, washed in saline, flash frozen, lyophilized, and stored until use at 4 °C.

### Characterization of microparticles and materials

BP content in PLGA particles was determined by dissolving particles in acetonitrile (ACN) and performing ultra performance liquid chromatography (UPLC). Residual sodium chloride (NaCl) in the formulations, which did not dissolve in ACN, was weighed to approximate salt content. Material decomposition characteristics and NaCl content were also determined by thermogravimetric analysis (TGA), which monitors mass changes in samples as the temperature varies. Morphology of particles was determined using scanning electron microscopy (SEM). Refer to Supplemental Method 4 for more details.

### *In vitro* evaluation of BP release

*In vitro* release studies were performed using a dialysis method^14^ (Supplemental Method 5). For PLGA microparticle/matrix formulations, 40 mg of PLGA microparticles and 50 mg of Gelfoam® were weighed into a small container, and combined with 150 µL of diH_2_O, resulting in a final salt concentration of 0.9 wt% NaCl. For samples without matrix, 40 mg of PLGA microparticles were directly suspended with 1 mL of 1X PBS. In both cases, the ratio of BP-PLGA microparticles to non-BP-PLGA microparticles (40 mg total of microparticles) was varied to achieve predicted dosing of BP (Supplemental Table 1). For all formulations that contained matrix, samples were mixed by hand to form a moldable bolus, as shown in the exemplary digital camera image in Supplemental Fig. 2. Periodically, the dialysis tubing was transferred to fresh buffer preconditioned at 37 °C. Buffer samples were stored at 4 °C until UHPLC was used to analyze *in-vitro* release of BP. To assess diffusion of free drug through the matrix, 1 mg of unencapsulated BP (free BP as the BP-HCl form) was added directly to either 50 mg or 100 mg of matrix (Gelfoam®, a water-insoluble, hemostatic sponge comprising of purified procine skin geletin). UHPLC analysis of released BP was performed by a modified version of a previously described method^13,15^ (Supplemental Method 6).

### Animals

Forty male and female Sprague Dawley rats, weighing 200 g–430 g, were bred in-house from animals originally received from Charles River. Rats of the same sex and treatment group were pair-housed and maintained in a climate-controlled facility under a 12-hour light/dark cycle. Rats had *ad libitum* access to water and food until the 3^rd^ day of handling and habituation, at which point food was restricted per rat per day to 20 g Harlan Laboratory Chow. This was determined to be in excess of the daily caloric requirement^16^. All procedures were approved by the UNC Institutional Animal Care and Use Committee, and the experiments were performed in accordance with the ARRIVE guidelines^17^, and the Committee for Research and Ethical Issues of the International Association of the Study of Pain.

### Food preparation

Each cage of pair-housed rats was supplied daily with 20 g feed in alternate sides of a dual food hopper, and 20 g feed soaked in water and provided as moist mush in a receptacle on the cage floor. Provision of moist mush mimics current recommended standard of human care to eat soft food immediately following tooth extraction^18^.

### Surgical procedure

The 1^st^ and 2^nd^ mandibular molars on the same facial side were extracted, with the side of extraction alternating between groups to control for chewing bias. Animals were anesthetized with an intraperitoneal (i.p.) injection of a 40 mg/mL ketamine hydrochloride, 8 mg/mL xylazine, and 0.5 mg/mL acepromazine maleate mix. Eyes were lubricated with ophthalmic ointment, a heat source was provided, and 2 mL 0.9% sodium chloride was administered subcutaneously (s.c.). Once a surgical plane of anesthesia was confirmed, 2 mg/kg BP was administered s.c. in the facial area near the extraction site as the ‘standard of care’ analgesic^4^. Following this, the lip and tongue were gently pulled back, the gum cut away from the molars with a #22 scalpel blade, and the roots exposed. The teeth were loosened by gentle backwards and forwards rocking, and removed with a standard Blumenthal rongeur. Blood and saliva were washed out of the mouth, the tooth socket dried, and the hemostatic composite packed down into the socket.

### *In vivo* Gelfoam^®^ formulations

Five separate groups of males and females, with 4 animals randomly assigned per group, received one of the following formulation complexes: (1) Gelfoam^®^ vehicle (GelVeh), (2) 2 mg/ml s.c. BP followed by Gelfoam^®^ vehicle (BP+GelVeh), (3) 2 mg/ml s.c. BP followed by Gelfoam^®^ complex with 1 mg/ml BP (BP+GelBP1), (4) 2 mg/ml s.c. BP followed by Gelfoam^®^ complex with 0.5 mg/ml BP (BP+GelBP.5), and (5) 2 mg/ml s.c. BP followed by Gelfoam^®^ complex with 0.25 mg/ml BP (BP+GelBP.25) (Supplemental Table 2) (Supplemental Method 7).

### Behavioral phenotyping

Rats were handled and habituated to the testing environment at the same time of day for 4 days prior to the experiment. Food consumption and body weight were measured prior to and on days 1–7 following molar extraction using a digital scale.

Subsequently, sensitivity to mechanical and thermal cold stimuli was determined prior to and on days 1, 2, 3, 5, and 7 following molar extraction, with measurements collected for both the treated (ipsilateral) and untreated (contralateral) orofacial sites. The mechanical set-up was adapted from that established by Krzyzanowska and colleagues^19^ (Supplemental Method 8), where the number of withdrawals was recorded at each time point. Mechanical hyperalgesia was defined as an increase in the number of withdrawals in response to the noxious stimulus.

Thermal cold sensitivity was measured by placing a cryostim probe maintained at 4 °C, to the vibrissal pad for a maximum of 20 seconds, or until the rat withdrew from the stimulus^20,21^. Withdrawal latencies were recorded in duplicate on ipsilateral and contralateral sides. If the second latency recorded was not within ±4 seconds of the first, a third measure was recorded. The 2 latencies closest in value were averaged to determine overall withdrawal latency. Thermal hyperalgesia was defined as a decrease in withdrawal latency in response to the cryostim probe.

### Statistical analyses

Sample sizes were selected based on their ability in previous, similarly structured rat studies to accurately demonstrate behavioral differences between groups^22–24^. Behavioral phenotyping data were analyzed by 2-way analysis of variance (ANOVA) to examine group differences over time. Mechanical and thermal sensitivity data were further analyzed by 1-way ANOVA to evaluate group differences on day 1 following tooth extraction, as this was the time of peak hyperalgesia. *Post-hoc* comparisons were performed using the Tukey’s test correcting for multiple comparisons. For all analyses, statistical significance was defined as P ≤ 0.05. All statistical analyses were performed using GraphPad Prism.

### Data availability

The datasets generated during and/or analysed during the current study are available from the corresponding author on reasonable request.

## Results

### Development and characterization of sustained release BP formulations

**Figure 2:**
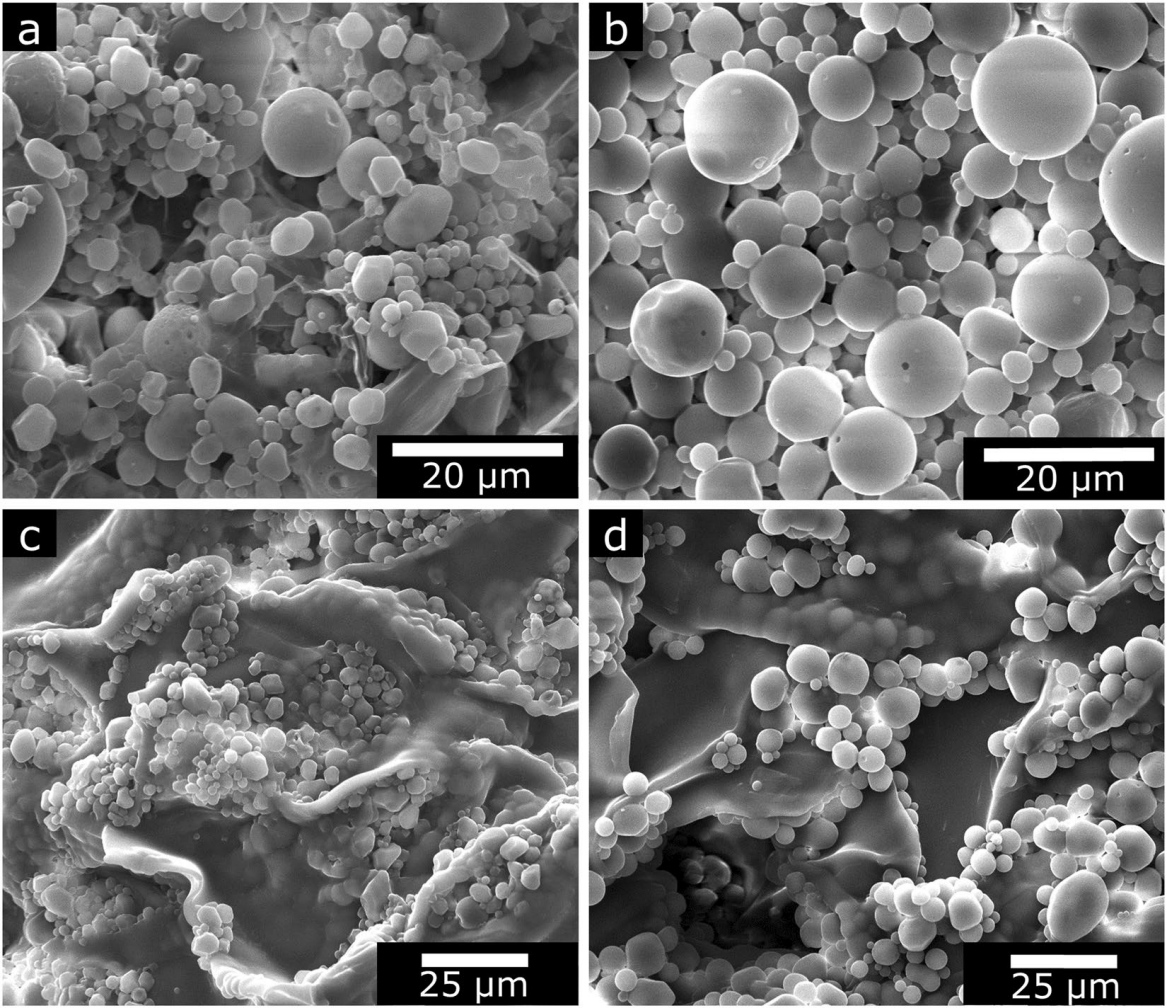
SEM images of (**a**) drug-free PLGA microparticles, (**b**) BP-PLGA microparticles, (**c**) drug-free PLGA microparticles + Gelfoam®, and (**d**) BP-PLGA microparticles + Gelfoam®.

In this report, we utilized the solvent evaporation method to encapsulate BP within PLGA microparticles, as previously reported^13^. To support this approach, BP-HCl was first converted to the base form^12,25^, and using PLGA with a molecular weight of 7–17 kDa, microparticles were generated that exhibited a smooth and predominantly spherical morphology, with diameters between approximately 1–20 µm. As shown by the SEM images (Fig. 2a,b), no appreciable morphological differences exist between BP-PLGA microparticles and microparticles without BP. To further characterize the samples, TGA studies were performed using the microparticles, the BP-Base, and the neat PLGA starting material (Supplemental Fig. 3). For the microparticles, the TGA curves were similar between BP-PLGA microparticles and the drug-free PLGA microparticles; the weight loss plateaued at 19 wt% ± 2 wt% at 600 °C for both formulations, with the plateau corresponding to the remaining NaCl in the formulations. However, the BP-PLGA microparticles showed an earlier drop in weight (5% weight loss at 240 °C), as compared to drug-free PLGA microparticles (1% weight loss at 240 °C). This drop in weight at 240 °C for BP-PLGA microparticles suggests loss of drug from the microparticles in agreement with the lower onset of decomposition temperature for BP-Base (210 ± 2 °C). Neat PLGA starting material showed two regions in the decomposition profile suggesting the possibility of distinct degradation products^26^.

To ascertain the BP loading efficiency, six replicate batches of BP-PLGA microparticles were prepared, washed, lyophilized, and subsequently quantified using UPLC. The drug loading of BP within the microparticles was 5 wt% ± 1 wt%, which was adequate for the targeted dosing requirements for the subsequent *in vivo* studies.

To achieve a long-acting formulation for placement within an extraction site, BP-PLGA microparticles were combined with a hemostat derived from porcine skin gelatin. Gelfoam® readily combined with the PLGA microparticles through physical mixing, as shown in Fig. 2c,d. Although the microparticles were not chemically linked to the hemostat, it was anticipated that the hemostat would spatially confine the microparticles, while simultaneously allowing the diffusion of BP. To test the diffusion of free BP through the hemostat, the material (i.e., free BP mixed with hemostat) was submerged in physiological buffer (1X PBS), and the released BP measured over time. As anticipated, the hemostat materials did not impede the diffusion of BP, such that the majority of free BP was released from the hemostat over 24 hours (Supplemental Fig. 4).

**Figure 3:**
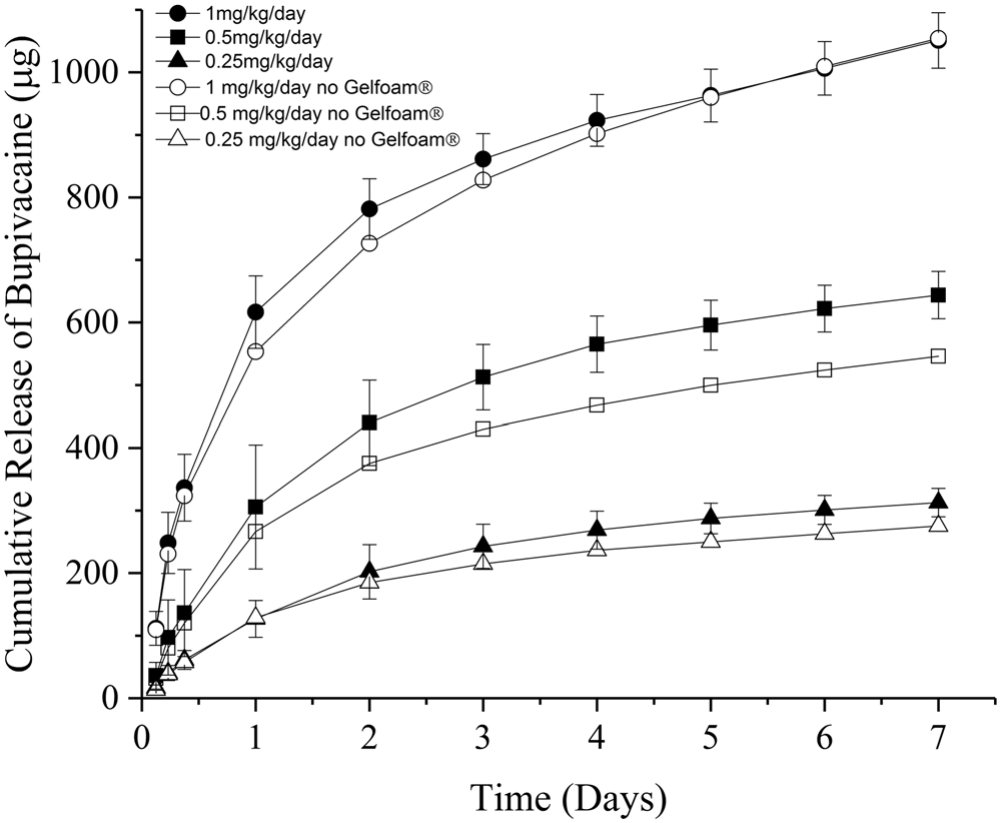
*In vitro* release of BP from GelBP (PLGA microparticles + Gelfoam® (filled markers)) or PLGA microparticles without Gelfoam® (open markers). All samples were incubated at 37 ˚C in 1X PBS pH = 7.2. Experiments containing the PLGA particles in Gelfoam® were performed in triplicate.

A series of *in vitro* studies were performed to further understand the release kinetics of BP from microparticles. Using the dialysis method, samples were added to dialysis tubing, submerged in physiological saline buffer (1X PBS at pH 7.2) at 37 °C, and the buffer was periodically quantified for release of BP. Four GelBP formulations were developed, with each formulation containing a specific weight ratio of BP-PLGA microparticles to drug-free PLGA particles, which were combined with Gelfoam (Supplemental Table 1). To accommodate the approximate volume within a dental cavity of a rat, a total of 40 mg of microparticles and 50 mg of Gelfoam® were combined, resulting in a total quantity of BP at 1.4 mg, 0.7 mg, 0.35 mg, and 0 mg. Figure 3 shows the BP release profiles over time for each formulation. The corresponding samples without Gelfoam® were also tested to evaluate the effect of Gelfoam on the BP release kinetics. BP was similarly released from PLGA microparticles, irrespective of the presence of Gelfoam®, in agreement with the results in Supplemental Fig. 4.

### *In vivo* studies

**Figure 4:**
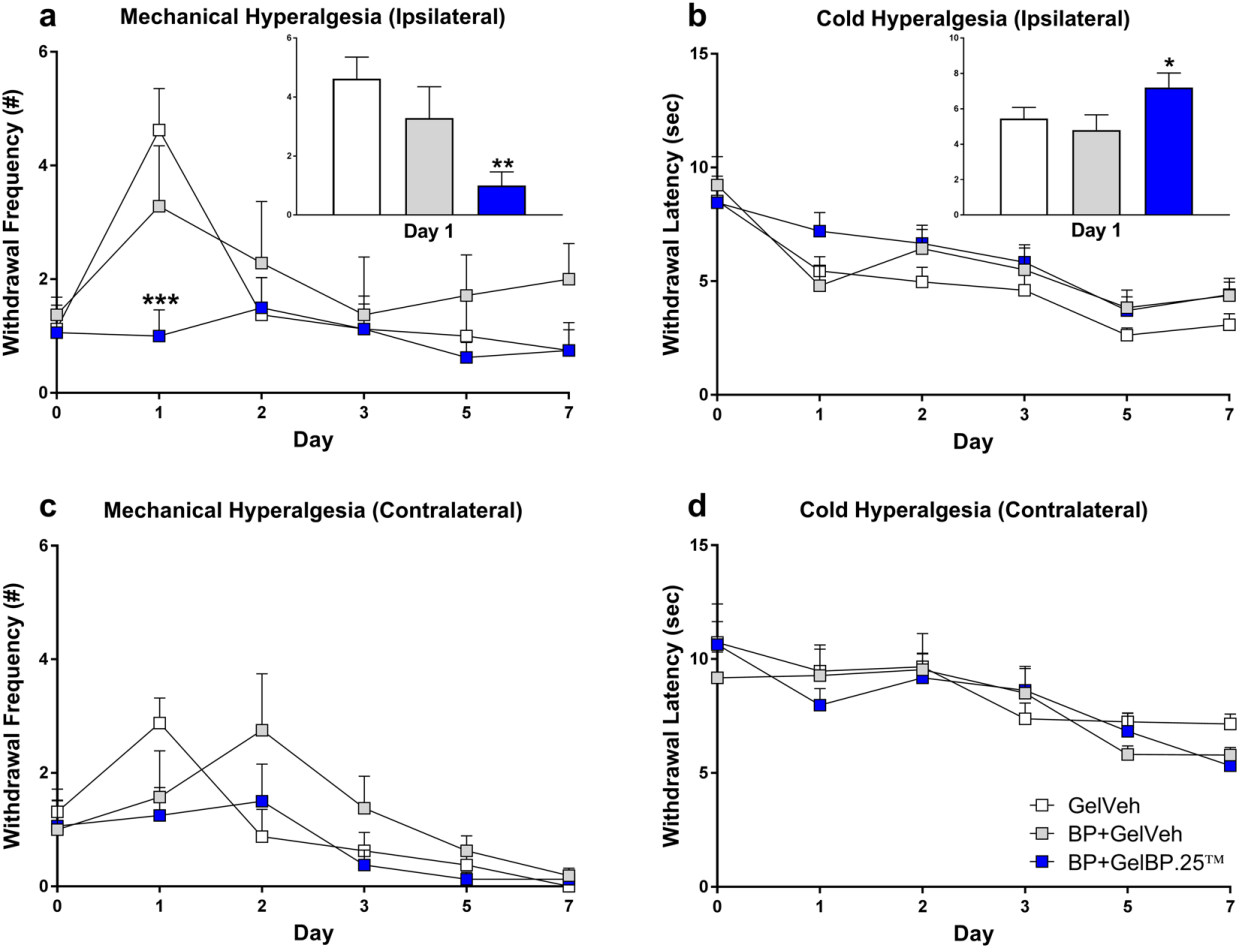
GelBP outperforms acute BP in blocking post-surgical dental pain. Following tooth extraction, rats in the GelVeh control group exhibited mechanical hyperalgesia (**a**) and cold hyperalgesia (**b**), that peaked on day 1 following surgery. Administration of BP+GelBP.25™, but not BP+GelVeh, prevented hypersensitivity to mechanical and thermal stimuli. A trend towards contralateral mechanical sensitivity was observed among rats receiving GelVeh or BP+GelVeh on day 1 or day 2, respectively, but not among those receiving BP+GelBP (**c**). No differences in contralateral cold sensitivity were observed between groups at any time point (**d**). Data are expressed as mean ± SEM. N = 8 (4 males + 4 females) per group. *P ≤ 0.05, **P ≤ 0.01, and ***P < 0.001 different from GelVeh.

The molar extraction procedure produced hypersensitivity to mechanical pressure and thermal cold. Behavioral responses were similar in males and females, so data were pooled. Rats in the GelVeh control group exhibited a 4-fold increase in withdrawal frequency to a punctate mechanical stimulus applied to the vibrissal pad ipsilateral to the tooth extraction site on day 1, indicative of mechanical hyperalgesia (Fig. 4a). Mechanical hyperalgesia was completely blocked by BP+GelBP.25, but not by BP+GelVeh (F_(2,21)_ = 5.370, P < 0.01). Rats in the GelVeh control group also exhibited a 5 second decrease in withdrawal latency to a thermal cold stimulus applied to the vibrissal pad ipsilateral to the tooth extraction site on day 1, indicative of cold hyperalgesia (Fig. 4b). Cold hyperalgesia was blocked by BP+GelBP.25, but not by BP+GelVeh (F_(2,21)_ = 3.296, P = 0.05). Formulations with higher BP (BP+GelBP.5 and BP+GelBP1) were ineffective in blocking mechanical or cold hyperalgesia (Supplemental Fig. 5a,b).

A trend towards contralateral mechanical sensitivity was observed among rats receiving GelVeh or BP+GelVeh on day 1 or day 2, respectively, but not among those receiving BP+GelBP.25, BP+GelBP.5, or BP+GelBP1 (Fig. 4c; Supplemental Fig. 5c). No differences in contralateral cold sensitivity were observed between groups at any time point (Fig. 4d; Supplemental Fig. 5d).

**Figure 5:**
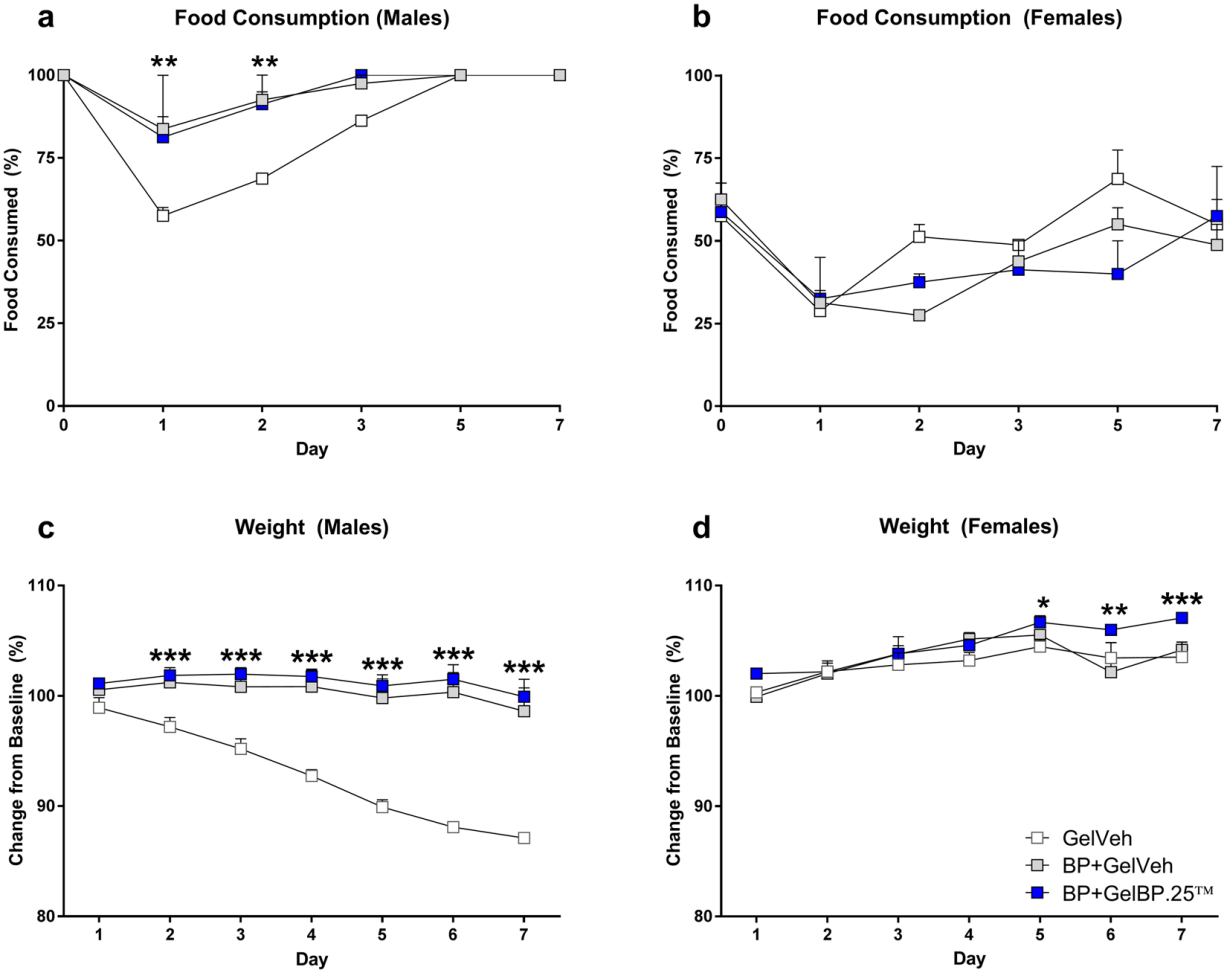
GelBP improves food consumption and weight following tooth extraction. Male (**a**) and female (**b**) rats receiving only GelVeh after tooth extraction exhibited decreased food consumption. Males receiving BP+GelBP.25 or BP+GelVeh maintained normal food consumption throughout the 7-day testing paradigm, though significant group differences were not observed in females. Among males, those receiving BP+GelBP.25 or BP+GelVeh maintained normal weight throughout the 7-day period (**c**). Among females, only those receiving BP+GelBP.25 maintained weight on days 5–7 (**d**). Data are expressed as mean ± SEM. N = 4 males or 4 females per group. **P ≤ 0.01 and ***P < 0.001 different from GelVeh.

The molar extraction procedure produced an ~40% decrease in food consumption in males (Fig. 5a) and females (Fig. 5b) in the GelVeh control group on day 1, which normalized by day 5. Decreased food consumption was associated with corresponding reductions in weight for males on days 2–7 (Fig. 5c), and to a lesser extent, females on days 6–7 (Fig. 5d). Of the three GelBP doses applied, optimal results were achieved with the lowest GelBP.25 dose. Among males, those receiving BP+GelBP.25™ or BP+GelVeh failed to exhibit alterations in food consumption (F_(6,18)_ = 9.45, P < 0.002) or weight (F_(6,18)_ = 49.93, P < 0.0001) throughout the 7-day testing paradigm compared to those receiving GelVeh. Among females, those receiving BP+GelBP.25™ exhibited greater weight gain on days 5–7 compared to those receiving GelVeh or BP+GelVeh (F_(6,18)_ = 3.04, P < 0.04), though no differences were observed in food consumption. BP+GelBP.5 and BP+GelBP1 did not improve food consumption in males or females, though males receiving BP+GelBP.5 exhibited normal weight gain throughout the 7-day testing paradigm (F_(6,18)_ = 74.81, P < 0.0001; Supplemental Fig. 6).

As described, the GelBP.25 formulation demonstrated optimal effects in preventing post-surgical pain (i.e., mechanical and cold hyperalgesia) and corresponding reductions in weight loss in both males and females.

## Discussion

We developed a novel platform for sustained delivery of anesthetic drug using drug-loaded microparticles embedded within a hemostat matrix. The formulation comprises a moldable putty for customized placement within an extraction site, and continuous delivery of BP to prevent post-operative dental pain over the course of several days. To the best of our knowledge, this is the first demonstration of a tailorable anesthetic formulation designed for an extraction cavity during management of post-operative dental pain.

PLGA microparticles were used as the carrier for BP, as PLGA offers excellent biodegradability, biocompatibility, and safety for microencapsulation and continuous delivery of therapeutic drugs^27^. While various approaches exist to fabricate PLGA microparticles for controlled release of drugs, we used solvent evaporation, owing to its compatilbity with encapsulation of BP and scale-up capabilities for producing larger batches of microparticles^13^.

The drug loading of BP within the microparticles was 5 wt% ± 1 wt%. This lower than anticipated loading efficiency may have resulted from factors concerning formulation parameters (e.g., drug/PLGA ratio, content of emulsifier) and processing conditions (e.g., washing protocol, stirring speed, solvent evaporation rate)^28,29^. The kinetic profile for BP release from the formulations followed a similar biphasic pattern in the absence and presence of Gelfoam® (Le Corre Estebe *et al*. 2002; Zhang Lu *et al*. 2008). The higher release within the first 24 hours, followed by subsequent tapering of release kinetics is compatible with the potential need for higher dosing of BP immediately post-surgery, when dental pain is prominent, and the requirement for less drug as the healing process begins.

The final GelBP formulations with three targeted dosing profiles (0.25, 0.5, and 1 mg/kg/day) were evaluated alongside acute subcutaneous BP injections (2 mg/kg) to determine their analgesic efficacy in a rat model of tooth extraction pain. GelBP outperformed acute BP in blocking post-surgical dental pain, with administration of the 0.25 mg/kg GelBP dose preventing hypersensitivity to both mechanical and thermal cold stimuli. Further, application of the 0.25 mg/kg GelBP prevented post-surgical weight loss associated with decreased food consumption. Thus, compared to the acute ‘standard of care’ injections, GelBP improved dental pain management by prolonging drug effectiveness.

Notably, increasing the concentration of BP did not produce a corresponding increase in analgesia. While the reason for this is unclear, our finding is in line with the results from several clinical studies that compared different doses of bupivacaine or levobupivacaine, and found that continuous infusion of the lowest concentration provided optimal pain management following ligament repair and abdominal surgery^30–33^. Thus, it is possible that BP-PLGA concentrations below 0.25 mg/kg in Gelfoam and subcutaneous BP doses below 2 mg/kg are even more effective in preventing post-surgical pain. In future studies, we plan to test a wider range of BP concentrations, particularly at the low end, and PLGA formulations in order to determine the optimal formulation. Further, we plan to measure drug availability and metabolism in local and systemic tissues as well as corresponding changes in pain-relevant biomarkers in order to provide additional insight into performance of this product.

This or a similar platform design may be used in conjunction with alternatively active pharmaceutical agents. For example, microparticles embedded in Gelfoam could be loaded with antibiotics or vascoconstrictor agents, and applied to extraction sites to prevent infection and promote healing. Additionally, this design could be adapted for delivery of anesthetic agents to other sites, such as topical wounds. Future studies are required to optimize new formulations for additional applications or drug systems.

## Conclusion

This report describes a new formulation for treatment of dental pain comprising BP-PLGA microparticles, which were embedded within a hemostatic matrix material. The hemostatic agent, Gelfoam®, was successfully combined with the BP-PLGA microparticles and used for *in vivo* studies in the rat animal model. *In vitro* studies showed sustained release of BP, which exhibited faster release kinetics early in the experiment that tapered over seven days. Subsequent pre-clinical studies demonstrated that application of the GelBP.25 formulation to molar extraction sites prevented the development of post-surgical dental pain and corresponding reductions in weight loss. This new formulation for anesthesia that resides within an extraction socket for a sustained dosing regimen and hemostatic function offers a novel approach for controlling post-operative dental pain.

## Electronic supplementary material

Supplemental Materials
