# Supplementary material for: A moldable sustained release bupivacaine formulation for tailored treatment of postoperative dental pain
Source: Sci Rep. 2018 Aug 15;8:12172. doi: 10.1038/s41598-018-29696-w (PMC6093872; doi:10.1038/s41598-018-29696-w)

A moldable sustained release bupivacaine formulation for tailored treatment of postoperative dental pain

Sarah D. Shepherd[a], Sandra C. O'Buckley[b], James M. Harrington[a], Laura G. Haines[a], Ginger D. Rothrock[a], Leah M. Johnson[a]*, and Andrea G. Nackley[b]*

Supplemental Materials and Methods

1. Conversion of BP-HCl to BP-base

Briefly, 25 mL of a sodium bicarbonate solution (2.6 g in 50 mL of diH_2O) was slowly added to a solution of 5 g of BP-HCl in 150 mL of diH_2O. After allowing the solution to stand for approximately 10 minutes, the Bupivacaine-base (BP-base) was extracted with dichloromethane three times (150 mL each), and dried over magnesium sulfate. Solvent was then extracted via rotary evaporation, and the resultant BP-base oil product was purified by precipitation and dried.

2. XRD analysis

BP-Base and BP-HCl were analyzed with a Shimadzu XRD-600 instrument using a Bragg-Brentano geometry with a scintillation detector using a monochromator. The instrument used a copper anode as the radiation source, and analysis parameters of 0.04 degree two-theta steps for a dwell time of 4 seconds each step over a range of 8-70 degrees two-theta at power settings of 45 kV and 40 mA. The beam was conditioned through fixed divergence and scatter slits of 0.5 degree width and a receiving slit of 0.3 degrees width to maximize intensity and reduce background.

3. Emulsion-solvent evaporation method

In a typical procedure, a solution comprising 500 mg of PLGA (ester terminated, 50:50 ratio of lactic acid to glycolic acid, 7-17K Da), and 105 mg of BP-base in 9 mL of methylene chloride was added to 100 mL of 100 mM Trizma-buffered aqueous solution of 0.5% poly-vinyl alcohol (6000 MW), and subsequently homogenized at ambient temperature for 60 seconds.

4. Characterization of microparticles and materials

Thermogravimetric analysis (TGA). TGA was used to monitor mass changes in samples as the temperature varied, providing insight into the material decomposition characteristics and content

(e.g., NaCl content). TGA measurements were performed with a TA Q50 instrument using a nitrogen gas atmosphere at a ramp rate of 10°C/min from 25°C to 600°C. All TGA measurement were performed in triplicate.

Scanning electron microscopy (SEM). SEM images were acquired to examine morphology of the particles and formulations. Samples were deposited on aluminum stubs, and sputter coated with a layer of gold/palladium, to create a charged surface. The particles were then examined using an FEI-brand SEM with an accelerating voltage between 5-20 kV under high vacuum.

5. Dialysis method

Briefly, each sample was placed in dialysis tubing (Spectra/Por Float-A-Lyzer, cellulose ester, molecular weight cutoff of 8000-10000), sealed, and incubated in physiological saline buffer (1X PBS, pH =7.2) while being stirred at 37°C.

6. UHPLC analysis

Chromatographic analysis was performed on a Waters ACQUITY UHPLC system equipped with a PDA detector set to 205 nm to quantify BP. A 0.10 mol/L phosphate buffer was prepared by dissolution of sodium dihydrogen phosphate monohydrate salt (98+%, ACS Reagent), and the pH adjusted to 4.00 by addition of 1% HCl solution (concentrated HCl, Trace Metals Grade, Fisher Scientific). ACN (≥99.9%, Sigma Aldrich) was used as the second eluent. Calibration standards were prepared by dissolution of solid BP acid and base forms to produce stock solutions, which were then further diluted into ACN to prepare the analytical standards at concentrations ranging from 0.68 – 55.5 µg/mL.

The acid form of BP was chromatographically separated on a Waters ACQUITY BEH C18 column (1.7 µm, 2.1 x 50 mm) by isocratic elution with 70% phosphate buffer/30% ACN at a flow rate of 0.60 mL/min. The base form of BP was chromatographically separated on a Waters ACQUITY UPLC HSS C18 SB column (1.8 µm, 2.1 x 50 mm) by isocratic elution with 55% phosphate buffer/45% ACN at a flow rate of 0.60 ml/min. Calibration standards were analyzed and peaks corresponding to BP were integrated, plotted against the concentration, and the calibration curve was calculated by linear least-squares regression without weighting. Calibration standards were

periodically reanalyzed to serve as quality control samples and monitor continuing system performance.

7. *In vivo* Gelfoam formulations

All formulations were supplied as a lyophilized complex of Gelfoam® + PLGA microparticles, with enough material for 4 animals, by RTI International to Duke investigators in numerically-coded tubes. All samples were stored at 4°C until preparation prior to use in 600 ul 0.9% NaCl. The respective complexes were gently mixed with a plastic spatula, rolled into a ball between gloved fingers, and divided into four equal parts. Each ¼ was applied to a different animal in the same group.

8. Mechanical behavioral phenotyping

In brief, animals were loosely restrained by placing them in a regular housing cage, divided down the middle, where escape was prevented by setting the lid loosely on the cage, allowing access to the vibrissal pads. Mechanical hyperalgesia was assessed using a 1.494 g von Frey filament applied to the vibrissal pad 10 times for a duration of 1 second, with an interstimulus interval of 1 second.

Supplemental Tables

Supplemental Table 1. Ratio of BP-PLGA microparticles to drug-free PLGA microparticles that were then combined with Gelfoam for use in the GelBP **formulations for controlled release of BP in vitro studies**

Targeted Dosing (mg/kg/day)	Ratio of PLGA Microparticles, BP-PLGA: drug-free PLGA
1	1:0
0.5	1:1
0.25	1:3

*Unless otherwise noted, each GelBP formulation contained a total of 40 mg microparticles + 50 mg Gelfoam®

Supplemental Table 2: Respective *in vivo* groups to determine the optimal BP+GelBP formulation for maximum analgesia

Group	s.c BP administered	GelBP formulation
1	X	0 mg/mL BP (vehicle only)
2	√	0 mg/mL BP (vehicle only)
3	√	1 mg/mL GelBP
4	√	0.5 mg/mL GelBP
5	√	0.25 mg/mL GelBP

Supplemental Figures

Supplemental Figure 1. **BP acid to BP-base conversion evaluated using X-Ray powder diffraction (XRD).** Diffraction profiles for (**a**) BP-HCL and (**b**) BP-Base are shown.

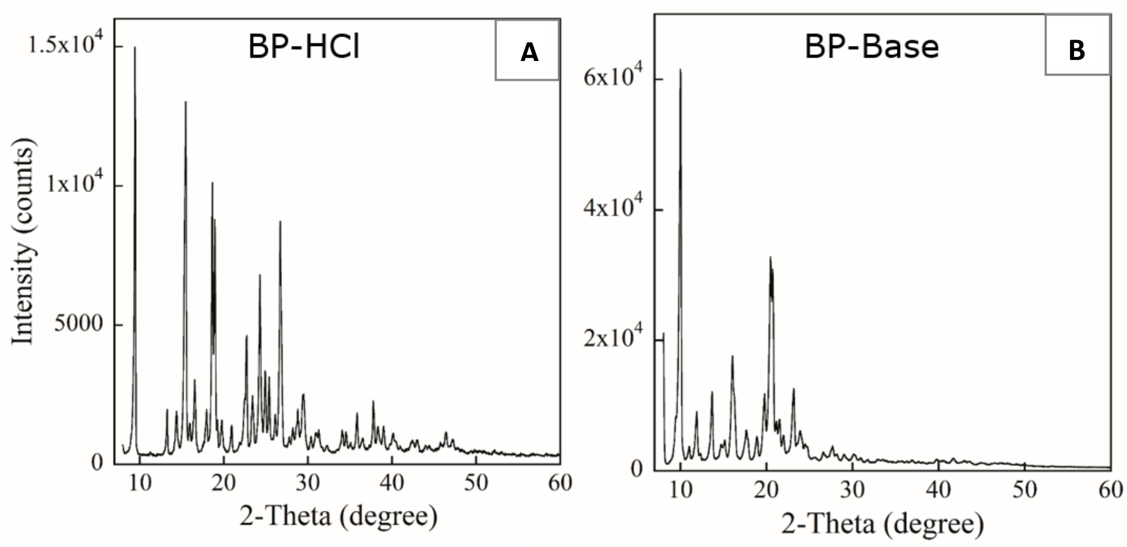

Supplemental Figure 2. **Digital camera image of hemostat matrix combined with PLGA microparticles.** The moldable bolus was formed by hand.

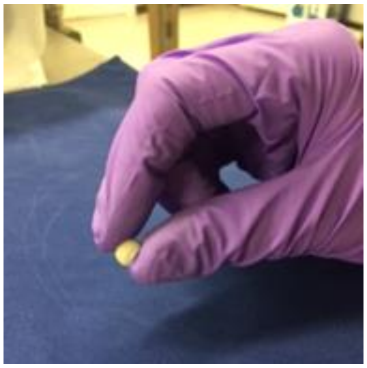

Supplemental Figure 3. **Thermogravimetric analysis of formulation and components.** Thermogravimetric analysis profiles showing % weight loss versus temperature for (**a**) PLGA microparticles without BP, (**b**) BP-PLGA microparticles (dashed line), (**c**) PLGA polymer (MW 7-17kDa) and (**d**) BP-Base. Each curve represents one TGA experiment.

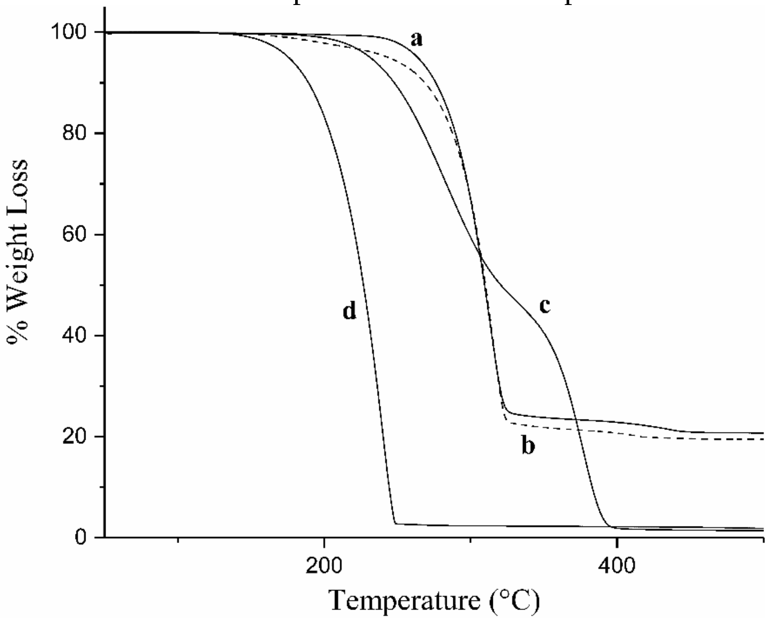

Supplemental Figure 4. **Release profile of unencapsulated BP from matrix material**. The majority of BP is released in the first 24 hours. 1mg of free BP-HCl in 0.9 wt/vol% NaCl was combined with Gelfoam® (at either 50 mg or 100 mg of matrix).

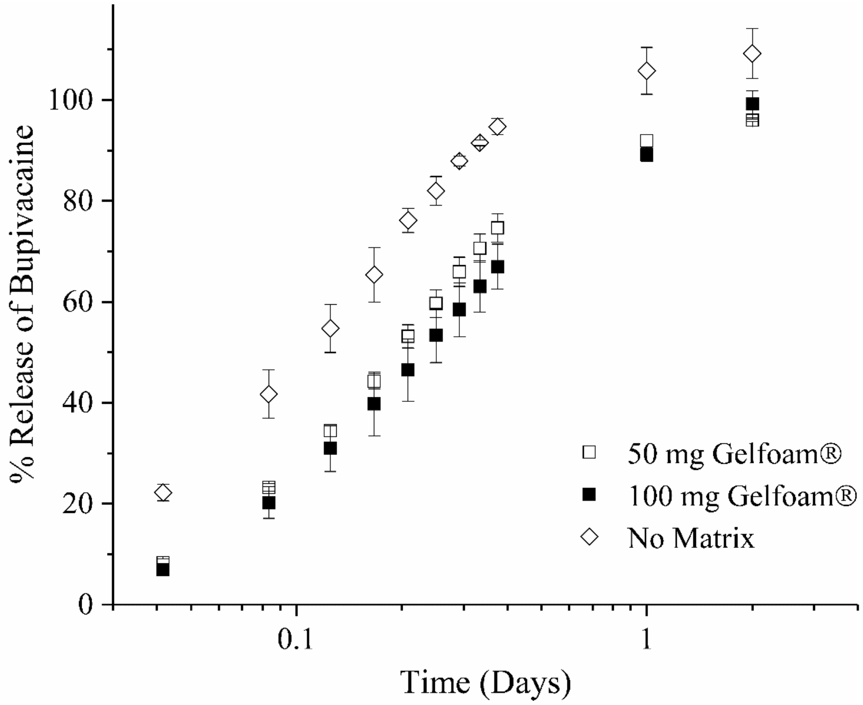

Supplemental Figure 5. Effect of GelBP.5 and GelBP1 on post-surgical dental pain. Following tooth extraction, rats in the GelVeh control group exhibited mechanical hyperalgesia (**a**) and cold hyperalgesia (**b**), that peaked on day 1 following surgery. Administration of BP+GelVeh, BP+GelBP.5, or BP+GelBP1 did not prevent hypersensitivity to mechanical and thermal stimuli. No differences in contralateral mechanical (**c**) or cold sensitivity were observed between groups at any time point (**d**). Data are expressed as mean ± SEM. N= 8 (4 males + 4 females) per group.

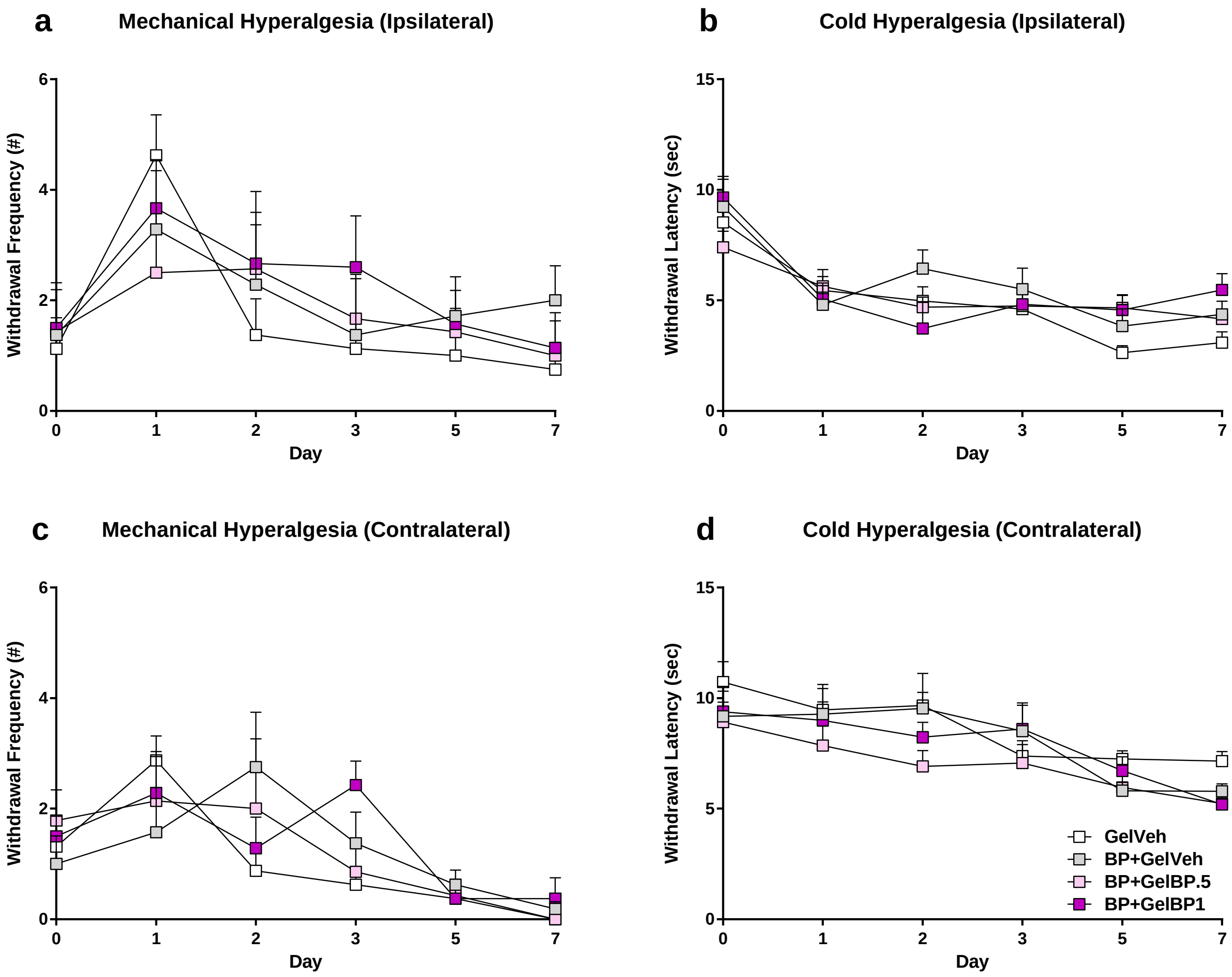

Supplemental Figure 6. **Effect of GelBP.5 and GelBP1 on food consumption and weight following tooth extraction.** Male (**a**) and female (**b**) rats receiving only GelVeh prior to tooth extraction exhibited decreased food consumption. Males receiving BP+GelVeh, but not BP+GelBP.5 or BP+GelBP1, maintained normal food consumption throughout the 7-day testing paradigm, though significant group differences were not observed in females. Among males, those receiving BP+GelBP.5 or BP+GelVeh maintained normal weight throughout the 7-day period (**c**). Among females, no group differences were observed (**d**). Data are expressed as mean ± SEM. N= 4 males or 4 females per group. *P ≤ 0.05, **P ≤ 0.01 and ***P < 0.001 different from GelVeh.

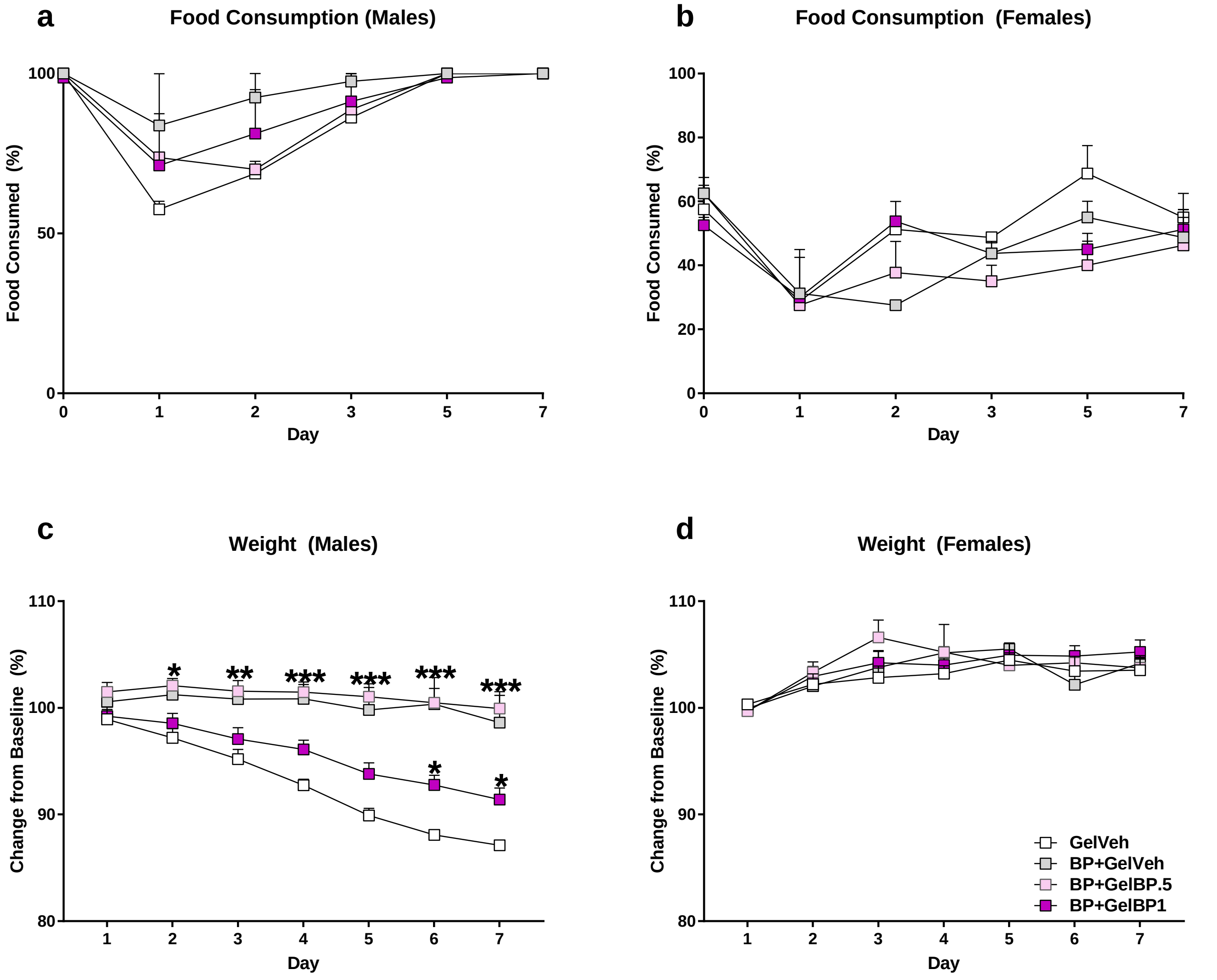

Supplement: Supplementary file 1 — Supplemental Materials [file 41598_2018_29696_MOESM1_ESM.pdf]
